# The impact of kidney function on plasma neurofilament light and phospho-tau 181 in a community-based cohort: the Shanghai Aging Study

**DOI:** 10.1186/s13195-024-01401-2

**Published:** 2024-02-12

**Authors:** Jie Wu, Zhenxu Xiao, Mengjing Wang, Wanqing Wu, Xiaoxi Ma, Xiaoniu Liang, Li Zheng, Saineng Ding, Jianfeng Luo, Yang Cao, Zhen Hong, Jing Chen, Qianhua Zhao, Ding Ding

**Affiliations:** 1 Institute of Neurology, Huashan Hospital, Fudan University, Shanghai, China; 2 National Clinical Research Center for Aging and Medicine, Huashan Hospital, Fudan University, Shanghai, China; 3 National Center for Neurological Disorders, Huashan Hospital, Fudan University, Shanghai, China; 4 Departemnt of Nephrology, National Clinical Research Center for Aging and Medicine, Huashan Hospital, Fudan University, Shanghai, China; 5 Department of Biostatistics, School of Public Health, Fudan University, Shanghai, China; 6 Clinical Epidemiology and Biostatistics, School of Medical Sciences, Örebro University, 70182 Örebro, Sweden; 7 MOE Frontiers Center for Brain Science, Fudan University, Shanghai, China

**Keywords:** Phospho-tau181, Neurofilament light, Comorbid conditions, Renal function, Glomerular filter rate

## Abstract

**Background:**

The blood-based biomarkers are approaching the clinical practice of Alzheimer’s disease (AD). Chronic kidney disease (CKD) has a potential confounding effect on peripheral protein levels. It is essential to characterize the impact of renal function on AD markers.

**Methods:**

Plasma phospho-tau181 (P-tau181), and neurofilament light (NfL) were assayed via the Simoa HD-X platform in 1189 dementia-free participants from the Shanghai Aging Study (SAS). The estimated glomerular filter rate (eGFR) was calculated. The association between renal function and blood NfL, P-tau181 was analyzed. An analysis of interactions between various demographic and comorbid factors and eGFR was conducted.

**Results:**

The eGFR levels were negatively associated with plasma concentrations of NfL and P-tau181 (*B* = − 0.19, 95% CI − 0.224 to − 0.156, *P* < 0.001; *B* = − 0.009, 95% CI − 0.013 to -0.005, *P* < 0.001, respectively). After adjusting for demographic characteristics and comorbid diseases, eGFR remained significantly correlated with plasma NfL (*B* = − 0.010, 95% CI − 0.133 to − 0.068, *P* < 0.001), but not with P-tau181 (*B* = − 0.003, 95% CI − 0.007 to 0.001, *P* = 0.194). A significant interaction between age and eGFR was found for plasma NfL (*P*_interaction_ < 0.001). In participants ≥ 70 years and with eGFR < 60 ml/min/1.73 m^2^, the correlation between eGFR and plasma NfL was significantly remarkable (*B* = − 0.790, 95% CI − 1.026 to − 0,554, *P* < 0.001).

**Conclusions:**

Considering renal function and age is crucial when interpreting AD biomarkers in the general aging population.

**Supplementary Information:**

The online version contains supplementary material available at 10.1186/s13195-024-01401-2.

## Background

Alzheimer’s disease (AD) is the leading cause of dementia [1]. Recently, blood-based biomarkers have been established as a non-invasive and cost-effective measure in AD, for early diagnosis [2], differential diagnosis [3], outcome prediction [4, 5], and longitudinal disease monitoring [6, 7]. Plasma neurofilament light chain (NfL) and phospho-tau181 (P-tau181) are promising AD biomarkers. The former is a biomarker of axonal injury [8], and the latter is one of the biomarkers of tau pathology [9]. Some studies have preliminarily indicated that the levels of plasma biomarkers might be influenced by various factors, including demographic features, comorbid diseases, and renal and liver function [10–12]. As blood-based biomarkers approach clinical practice, it is essential to clarify these factors and characterize the potential impact before defining the reference range in the general population.

Chronic kidney disease (CKD) is common in the aging population, with a prevalence of 38% among those aged ≥ 70 years in the USA [13]. Since reduced renal function can affect the clearance of substances in peripheral blood, renal function should be considered when exploring the reference range for blood-based markers. Previous literature has reported associations between medical comorbidities and AD plasma biomarkers. Several studies suggested that renal function indicators and medical history of CKD were associated with higher plasma total-tau, Aβ40, Aβ42 [14, 15], NfL [16–18], and P-tau181 levels [18–22]. However, a significant portion of the previous studies were hospital-based [14, 20], not including the Asian population, or lacked the data of glomerular filtration rate (GFR), a precise measure of kidney function [19, 23]. An Asian population-based study with objective assays, comprehensively examining the association between GFR and plasma AD markers, is urgently needed.

Based on a previously well-established population-based cohort, the Shanghai Aging Study (SAS), we aimed to investigate the association of estimated GFR (eGFR) with plasma NfL and P-tau181 levels. We also endeavored to identify potential demographic factors and comorbid conditions that could modify the association. This study will deepen our understanding of blood AD biomarkers and pave the way for their widespread implementation, both in clinical practice and community screening.

## Methods

### Study participants

The SAS was a community-based longitudinal cohort study that aimed to establish a prospective cohort to enumerate the prevalence, incidence, and risk factors for dementia and mild cognitive impairment (MCI) among residents in downtown Shanghai, China [24]. The inclusion criteria of SAS were described previously [24]. In the current study, the correlation of renal function with plasma NfL and P-tau181 was investigated by testing the blood samples and analyzing data of SAS at the baseline. Therefore, we included SAS participants who (1) were dementia-free at the baseline and (2) had blood samples collected and stored at the baseline for renal function and biomarkers testing.

This study was approved by the Medical Ethics Committee of Huashan Hospital, Fudan University (No. 2009–195). Written informed consent was obtained from all participants and their legal guardians.

### Demographics and comorbid variables

Demographic and lifestyle characteristics were acquired from study participants through a detailed questionnaire. Body height (cm) and weight (kg) were measured to calculate body mass index (BMI) (kg m^−2^). Education attainment was defined as the years of formal education at school. History of hypertension, diabetes, and stroke was confirmed by the medical records. Dementia was diagnosed according to the DSM-IV criteria [24]. Apolipoprotein E (APOE) genotype was determined [25], and APOE ε4-positive was defined as having at least one ε4 allele.

### Neuropsychological assessment and cognitive diagnosis

The neuropsychological test battery included the following: (1) Mini-Mental Status Examination (MMSE) [26], (2) Tail Making Test Parts A and B (TMTA, TMTB) [27], (3) Auditory Verbal Learning Test (AVLT) [28], (4) Stick Test (ST) [28], (5) Renminbi (official currency of China) Test translated from the EURO test [29], (6) Conflicting Instructions Task (Go/No-Go Task) [28], (7) modified Common Objects Sorting Test (MCOST) [28]. All tests were conducted in Chinese by professional psychometrists within 90 min. The raw scores for individual cognitive tests were transformed into *Z*-scores and averaged to evaluate five clinically significant cognitive domains, including memory, language, attention, visuospatial and executive function.

Participants with normal cognition (NC) had been confirmed cognitively intact through a detailed neuropsychological assessment. Meanwhile, MCI was diagnosed according to Petersen’s criteria [30]: (1) patients should be judged as impaired but not fulfilling diagnostic criteria for dementia; (2) functional activities of the person were mainly preserved, or at least that impairment is minimal.; and (3) the person should have evidence of cognitive decline, measured either by self and/or informant report in conjunction with deficits on objective cognitive tasks, and/or evidence of decline over time on objective neuropsychological tests.

### Measurement of serum creatinine and cystatin C

Fasting blood samples were collected from participants at baseline from January 2010 to September 2011. Samples were centrifuged at 1000 g, for 15 min (4 °C). Serum and plasma were aliquoted and immediately stored at -80 °C until the day of the test in November and December of 2020. No freeze-thawing occurred during the storage.

Serum creatinine and cystatin C were measured by Roche Cobas 8000 modular analyzer Series (Roche, Inc., Mannheim, Germany) with a particle-enhanced immunonephelometric assay (Roche, Tina-quant Cystatin C, Mannheim, Germany) and the enzymatic assay creatinine PlusVer.2 (Roche, Mannheim, Germany) on Cobas c702 and Cobas c501 platforms, respectively [5].

### Calculation of estimated GFR

Concerning the primary eGFR estimation, we adopted the creatinine–cystatin C combined equation of Chronic Kidney Disease Epidemiology Collaboration (CKD-EPI), which performed better than the creatinine- or cystatin C-only equation [31]. Estimated GFR was calculated by CKD-EPI equations as follows:

$$eGFR=135\times\min(Scr/\kappa,1)^{\alpha}\times\max(Scr/\kappa,1)^{-0.601}\times\min(Scys/0.8,1)^{-0.375}\times\max(Scys/0.8,1)^{-0.711}\times\ 0.995^{Age}[\times\ 0.969~if~female][\times\ 1.08~if~black]$$

Scr was serum creatinine, Scys was serum cystatin C, κ was 0.7 for females and 0.9 for males, α was -0.248 for females and -0.207 for males, min indicated the minimum of Scr/κ or 1, and max indicated the maximum of Scr/κ or 1. CKD was classified into 5 stages according to the 2012 Guideline by the Kidney Disease: Improving Global Outcomes (KDIGO-CKD) organization [32]: CKD-stage 1: eGFR ≥ 90 mL/min/1.73 m^2^; CKD-stage 2: eGFR 60–89 mL/min/1.73 m^2^; CKD-stage 3: eGFR 30–59 mL/min/1.73 m^2^; CKD-stage 4: eGFR 15–29 mL/min/1.73 m^2^; CKD-stage 5: eGFR < 15 mL/min/1.73 m^2^. Considering the limited number of patients in CKD stage 4 and 5, the participants in this cohort were divided into three groups: high eGFR group (CKD-stage 1), medium eGFR group (CKD-stage 2), and low eGFR group (CKD-stage ≥ 3).

### Assay of plasma NfL and P-tau181

Plasma NfL and P-tau181 were assayed in November and December of 2020 using ultra-sensitive Simoa technology on the automated Simoa HD-X platform (Quanterix, MA, USA). The P-tau181 V2 (Cat. No. 103714) and NfL (Cat No:103186) assay kits were procured from Quanterix and utilized in accordance with the manufacturer’s instructions. Plasma samples were diluted at a 1:4 ratio for all assays. All samples were analyzed using the same batch of kits. All the operators were blind to the participants’ cognitive diagnosis and clinical information.

### Statistical analysis

The data were summarized and shown as “frequency (percentage)” for categorical data and “median [interquartile range (IQR)]” for continuous data due to non-normal distribution. Differences across three eGFR groups were compared using chi-square or Fisher’s exact test (where appropriate) for categorical variables, and the Kruskal–Wallis test for continuous variables.

The Kruskal–Wallis test was used to measure plasma NfL and P-tau181 levels among three groups and the Bonferroni method was used for multiple comparisons between each two groups. Density plots were used to illustrate the distribution of plasma biomarker levels in participants with different cognition diagnoses and eGFR levels. Wilcoxon test was conducted to measure plasma biomarker levels and neuropsychological test scores among different cognition diagnostic groups and eGFR groups. Multiple linear regression models were used to determine the relationship of eGFR with plasma biomarkers, adjusting for age, sex, BMI, education years, APOE genotype, stroke, hypertension, and diabetes. A further interaction analysis was conducted to assess whether the associations of eGFR with plasma NfL and P-tau181 levels were modified by various demographic and comorbidities variables (i.e., age, sex, education years, APOE, BMI, stroke, hypertension, and diabetes) using linear regression models. Subgroup analysis was conducted to examine whether the correlation of eGFR with plasma NfL and P-tau181 varied in different demographic and comorbid subgroups using linear regression models. The Spearman correlation was conducted to calculate the association between renal function-related laboratory measures and plasma NfL and P-tau181 levels.

Data analysis was conducted using IBM SPSS statistics version 26 statistical software and programming language R (version 4.2.1). The violin plot was visualized by GraphPad Prism version 9.4.1 (681). All tests were 2-sided. Statistical significance was set at *P* < 0.05.

## Results

### Characteristics of the study participants

A total of 1189 dementia-free participants aged ≥ 60 years were included in this study, as shown in Table 1. One hundred and ninety-five (16%), 796 (67%), and 198 (17%) participants were assigned to high, medium, and low eGFR groups, respectively. The participants in the low eGFR group were the oldest (median age = 75.6 years, *P* < 0.001) and demonstrated the highest levels of urea, creatinine, cystatin C, and uric acid (all *P* < 0.001). No significant discrepancy was found in other variables among the three eGFR groups.

**Table 1:** Demographics and clinical characteristics of study participants

	**All participants *n* = 1189**	**eGFR ≥ 90 *n* = 195**	**60 ≤ eGFR < 90 *n* = 796**	**eGFR < 60 *n* = 198**	***P* value**
**Characteristics**	**Median (IQR) or *n* (%)**	**Median (IQR) or *n* (%)**	**Median (IQR) or *n* (%)**	**Median (IQR) or *n* (%)**	
**Sex, female**	644 (54.2)	117 (60.0)	422 (53.0)	105 (53.0)	0.202
**Age, years**	70.24 (64.32, 75.88)	65.21 (62.79, 69.91)	70.42 (64.68, 75.69)	75.56 (69.70, 79.83)	< 0.001
**Education, years**	12.00 (9.00, 15.00)	12.0 (9.00, 15.00)	12.00 (9.00, 15.00)	12.00 (9.00, 15.00)	0.767
**BMI, kg/m2**	24.68 (22.55, 27.05)	24.39 (22.44, 26.56)	24.61 (22.59, 27.07)	25.39 (22.96, 27.20)	0.227
***APOE ε*4 carrier**	202 (17.0)	31 (15.9)	140 (17.6)	31 (15.7)	0.735
**Stroke**	154 (13.0)	19 (9.8)	103 (12.9)	32 (16.2)	0.164
**Hypertension**	621 (52.2)	109 (55.9)	401 (50.4)	111 (56.1)	0.191
**Diabetes**	167 (14.1)	33 (16.9)	109 (13.7)	25 (12.6)	0.410
**MMSE**	29 (28.00, 30.00)	29.00 (28.00, 30.00)	29.00 (28.00, 30.00)	29.00 (27.00, 29.50)	0.007
**Domain-specific**					
**Memory**^a^	0.03 (− 0.67, 0.62)	0.24 (− 0.36, 0.73)	− 0.05 (− 0.60, 0.62)	− 0.11 (− 0.96, 0.41)	< 0.001
**Visuospatial ability**^a^	− 0.34 (− 0.75, 0.46)	0.06 (− 0.75, 0.46)	− 0.34 (− 0.75, 0.56)	− 0.34 (− 0.75, 0.46)	0.028
**Attention**^a^	0.016 (− 0.65, 0.56)	0.18 (− 0.40, 0.89)	− 0.03 (− 0.68, 0.56)	− 0.08 (− 0.94, 0.49)	< 0.001
**Executive function**^a^	0.21 (− 0.23, 0.57)	0.35 (− 0.01, 0.65)	0.20 (− 0.20, 0.56)	0.08 (− 0.56, 0.49)	< 0.001
**Language**^a^	0.46 (− 0.30, 0.85)	0.46 (− 0.30, 0.85)	0.46 (− 0.30, 0.85)	0.08 (− 0.68, 0.85)	0.018
**CysC (mg/l)**	1.00 (0.88, 1.14)	0.79 (0.73, 0.84)	1.00 (0.93, 1.09)	1.33 (1.22, 1.46)	< 0.001
**Creatinine (mg/dl)**	0.86 (0.72, 1.01)	0.68 (0.60, 0.76)	0.85 (0.75, 0.97)	1.13 (0.98, 1.27)	< 0.001
**Uric acid (mg/dl)**	334.00 (285.50, 388.00)	295.00 (254.00, 343.00)	331.50 (286.00, 379.00)	389.00 (325.75, 452.00)	< 0.001
**Plasma NfL (pg/ml)**	15.50 (11.83, 21.38)	12.27 (9.73, 16.73)	15.50 (11.94, 20.25)	20.70 (15.17, 28.25)	< 0.001
**Plasma P-tau181 (pg/ml)**	1.91 (1.49, 2.47)	1.74 (1.39, 2.20)	1.90 (1.50,2.44)	2.17 (1.65, 2.77)	< 0.001
**eGFR (ml/min/1.73 m**^**2**^**)**	74.78 (64.53, 85.55)	95.93 (92.42, 100.28)	74.84 (68.79, 82.07)	52.36 (48.00, 56.00)	< 0.001

*Abbreviations*: *APOE* apolipoprotein E, *BMI* body mass index, *MMSE* Mini-Mental State Examination, *NfL* neurofilament light chain, *P-tau181* tau phosphorylated at threonine 181, *CysC* cystatin C, *eGFR* estimated glomerular filtration rate, *IQR* interquartile range

^a^*Z* score transformed

### Plasma NfL and P-tau181 levels in different eGFR groups

**Fig. 1:**
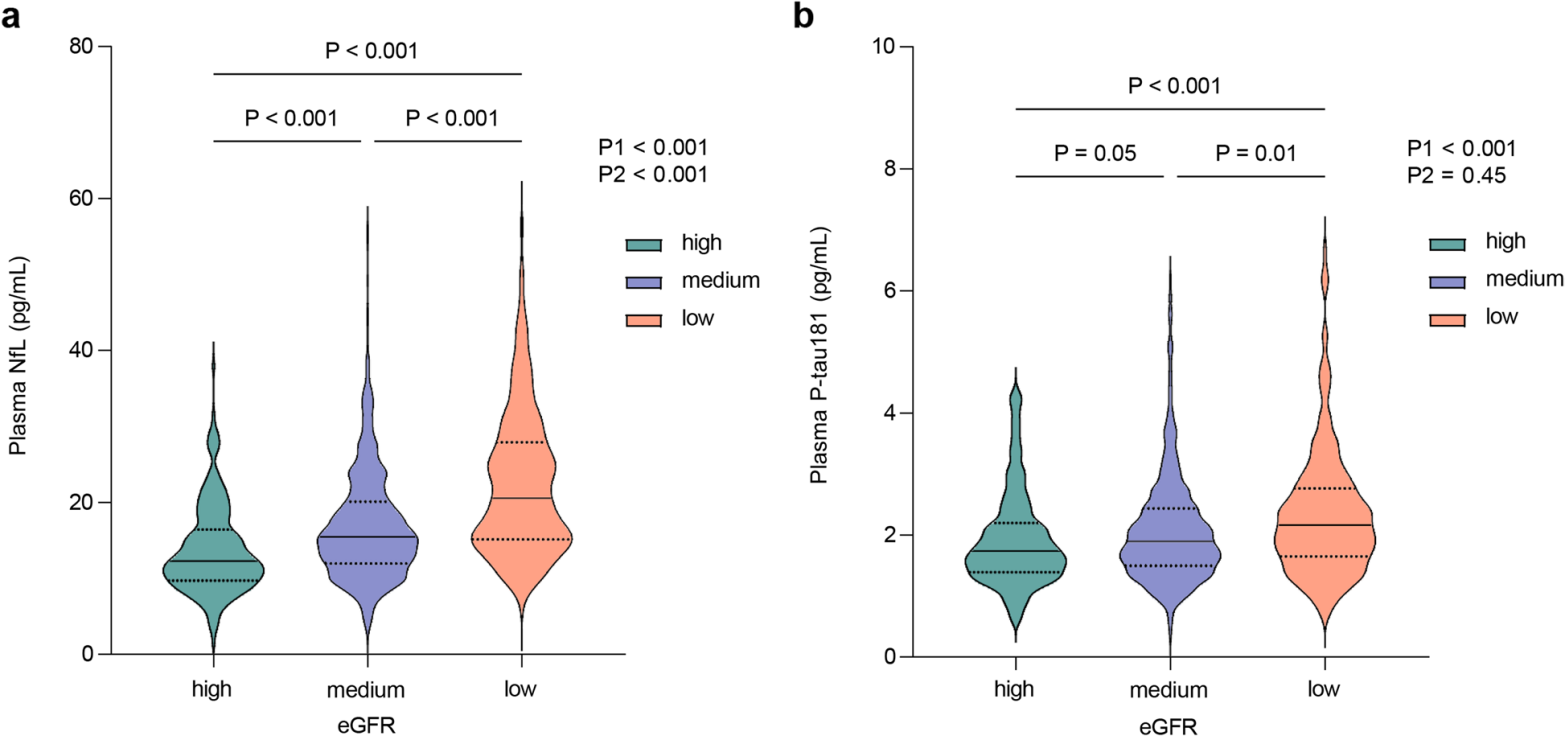
Plasma NfL and P-tau181 concentrations among different sub-groups by eGFR level. **a** plasma NfL concentration among different eGFR subgroups. **b** plasma P-tau181 concentration among different eGFR subgroups. All participants (*n* = 1189) were divided into 3 groups according to the KDIGO GFR classification, high eGFR group (eGFR ≥ 90 mL/min/1.73 m^2^), medium eGFR group (60 ≤ eGFR < 90 mL/min/1.73 m^2^) and low eGFR group (eGFR < 60 mL/min/1.73 m^2^). P1 and pairwise comparison was unadjusted. P2 was adjusted for sex, age, education, BMI, APOE genotype, hypertension, diabetes, and stroke. The violin plot showed data distribution. The solid lines represented the median, and the dashed lines represented the 25th and 75th quartiles. Abbreviations: NfL, neurofilament light chain; P-tau181, tau phosphorylated at threonine 181; eGFR, estimated glomerular filtration rate

A significant difference in plasma NfL level was found among three eGFR groups (median NfL = 12.3, 15.5, and 20.7 pg/mL in the high, medium, and low eGFR group, respectively, *P* < 0.001, all *P* post hoc comparisons < 0.001) (Fig. 1). The difference remained significant after adjusting for demographic and comorbidity characteristics including age, sex, BMI, education, APOE genotype, stroke, hypertension, and diabetes (*P* < 0.001). A significant difference in plasma P-tau181 level was observed among three eGFR groups (median = 1.7, 1.9, and 2.2 pg/mL in the high, medium, and low eGFR group, respectively, *P* < 0.001), but the difference was insignificant after adjusting for demographic and comorbidity characteristics (*P* = 0.45). Plasma P-tau181 levels in the medium (1.9 vs. 2.2 pg/mL, *P* = 0.001) and high eGFR groups (1.7 vs. 2.2 pg/mL, *P* < 0.001) were significantly lower than that in the low eGFR group. However, there was no significant difference in plasma P-tau181 concentration between the medium and high eGFR groups (1.9 vs. 1.7 pg/mL, *P* = 0.05).

### Overlap of plasma biomarker distribution between MCI and NC group with various eGFR levels

**Fig. 2:**
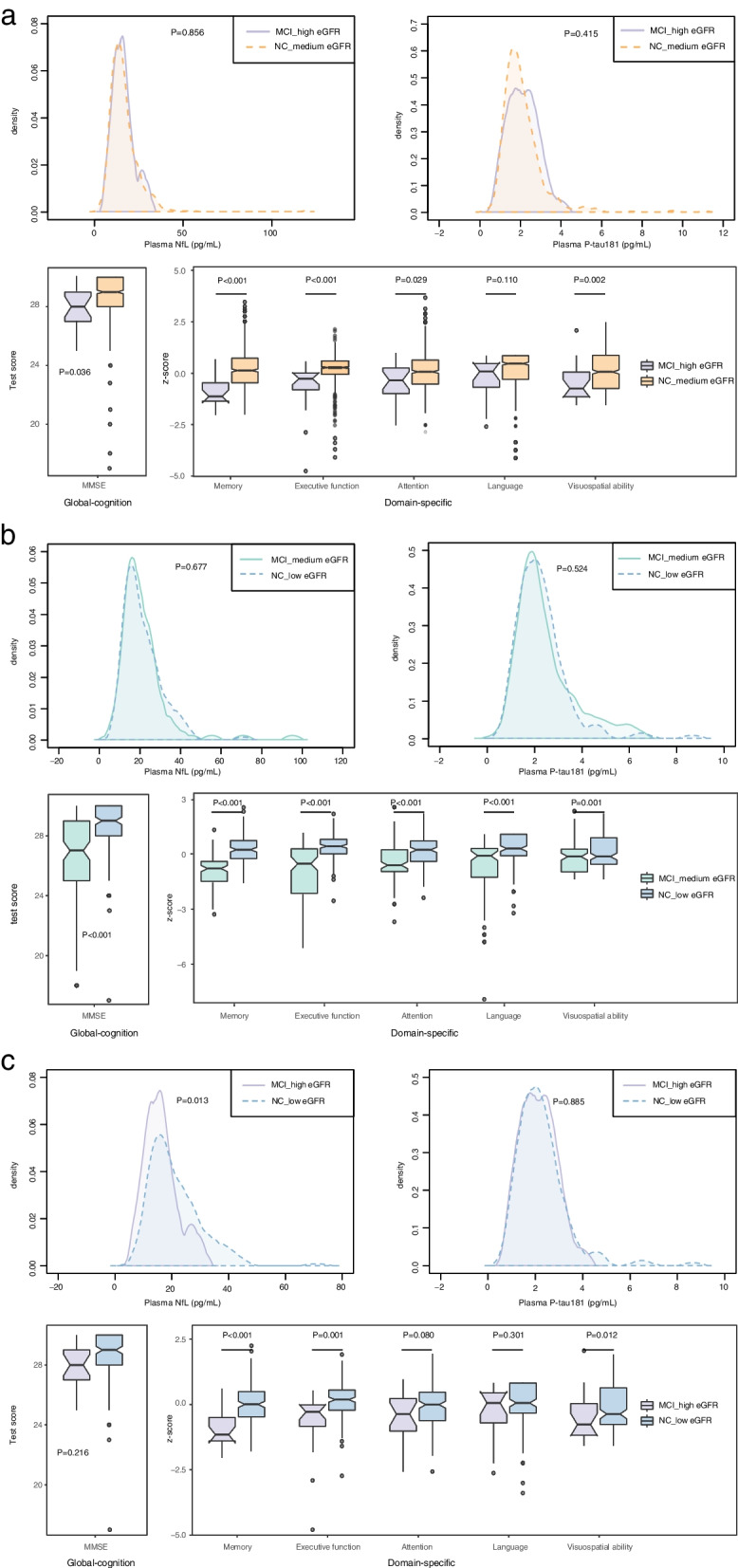
Overlap of plasma biomarker distribution between MCI and NC group with various eGFR levels. **a** Profile of plasma biomarker levels and neuropsychological test scores between MCI_high eGFR and NC_medium eGFR groups. **b** Profile of plasma biomarker levels and neuropsychological test scores between MCI_medium eGFR and NC_low eGFR groups. **c** Profile of plasma biomarker levels and neuropsychological test scores between MCI_medium eGFR and NC_low eGFR groups. The density plots show the distribution of plasma biomarker levels in different cognition and eGFR groups. The box plot showed the neuropsychological test scores in different cognition and eGFR groups. Abbreviations: NC, normal control; MCI, mild cognitive impairment; NfL, neurofilament light chain; P-tau181, tau phosphorylated at threonine 181; eGFR, estimated glomerular filtration rate; MMSE, Mini-Mental State Examination

To further illustrate the potential impact of renal function on the biomarkers regarding cognitive status, we used a density plot. Participants were divided into six subgroups based on their cognitive diagnosis and eGFR levels: NC with low-, medium- or high eGFR and MCI with low-, medium- or high eGFR levels. Although the subgroups were cognitively distinct from each other, they overlapped considerably in terms of plasma biomarker levels. Specifically, there was considerable overlap in NfL and P-tau181 levels between the MCI_high eGFR and NC_medium eGFR groups (Fig. 2a) and between the MCI_medium eGFR and NC_low eGFR group (Fig. 2b) (all *P* > 0.05). Additionally, P-tau181 levels overlapped between the MCI_high eGFR and NC_low eGFR groups (*P* = 0.885).

### Correlation of eGFR with plasma NfL and P-tau181 in multiple linear regression models

Table 2 showed that there was a negative correlation between eGFR and plasma NfL in both the unadjusted model 1 (*B* = − 0.190, 95% CI − 0.224 to − 0.156, *P* < 0.001) and the multivariate model 2 adjusting for age, sex, education, BMI, and APOE genotype (*B* = − 0.093, 95% CI − 0.126 to − 0.060, *P* < 0.001) and model 3 further adjusting for stroke, hypertension, and diabetes (*B* = − 0.010, 95% CI − 0.133 to − 0.068, *P* < 0.001). Although a negative correlation was identified between eGFR and plasma P-tau181 in basic model 1 (*B* = − 0.009, 95% CI − 0.013 to − 0.005, *P* < 0.001), insignificant correlations were shown in model 2 (*B* = − 0.003, 95% CI − 0.007 to 0.001, *P* = 0.21) and model 3 (*B* = − 0.003, 95% CI − 0.007 to 0.001, *P* = 0.19). Serum urea, Cystatin C, and creatinine showed significant correlations with plasma NfL (*r* = 0.194, *P* < 0.001; *r* = 0.291, *P* < 0.001; *r* = 0.166, *P* < 0.001, respectively) and P-tau181 levels (*r* = 0.133, *P* < 0.001; *r* = 0.148, *P* < 0.001; *r* = 0.122, *P* < 0.001, respectively). Uric acid was not significantly correlated with either NfL or P-tau181 (*r* = − 0.016, *P* = 0.58; *r* = 0.022, *P* = 0.45, respectively). Data was shown in Fig. S1.

**Table 2:** Multiple linear regression of plasma NfL, P-tau181 with eGFR

Plasma biomarkers	Model 1^a^			Model 2^b^			Model 3^c^		
	*B* (95% CI)	Standardized *B* (95% CI)	*P* value	*B* (95% CI)	Standardized *B* (95% CI)	*P* value	*B* (95% CI)	Standardized *B* (95% CI)	*P* value
NfL	− 0.190 (− 0.224, − 0.156)	− 0.304 (− 0.358, − 0.25)	< 0.001	− 0.093 (− 0.126, − 0.060)	− 0.149 (− 0.201, − 0.097)	< 0.001	− 0.100 (− 0.133, − 0.068)	− 0.16 (− 0.212, − 0.108)	< 0.001
P-tau181	− 0.009 (− 0.013, − 0.005)	− 0.127 (− 0.184, − 0.071)	< 0.001	− 0.003 (− 0.007, 0.001)	− 0.037 (− 0.095, 0.021)	0.214	− 0.003 (− 0.007, 0.001)	− 0.039 (− 0.097, 0.02)	0.194

*Abbreviations*: *NfL* neurofilament light chain, *P-tau181* tau phosphorylated at threonine

^a^Model 1 is a simple linear regression model

^b^In model 2, data were adjusted for demographic characteristics including gender, age, education, BMI, and APOE genotype

^c^In model 3, data were adjusted for demographic characteristics and comorbid diseases, eg. hypertension, diabetes, and stroke

### Subgroup comparison and interaction analysis

**Fig. 3:**
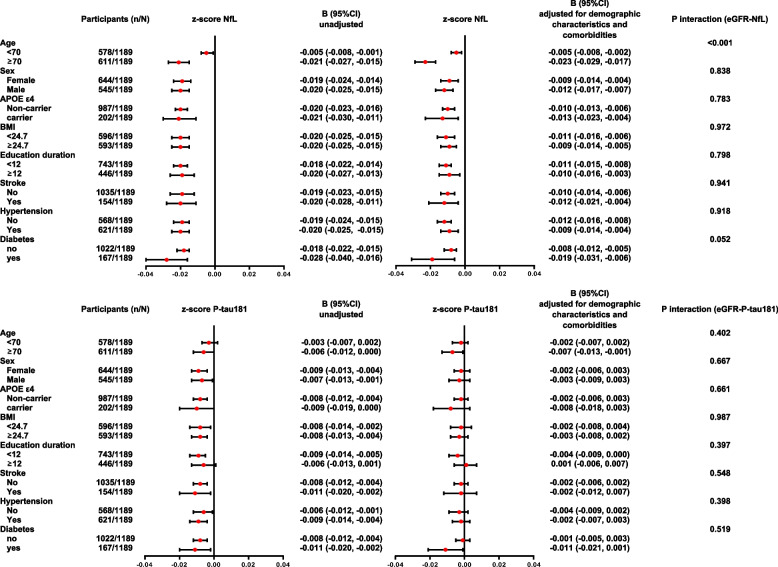
Forest plots of subgroups and interaction analysis. Forest plots illustrated the association between eGFR and plasma biomarker levels in different subgroups. The value of P interaction demonstrated whether the association between eGFR and plasma biomarker levels was modulated by demographic characteristics and co-morbid diseases. Since the concentrations of plasma NfL and P-tau181 differed by magnitude, the raw plasma level was z score transformed for parallel comparison between the coefficients. Means and 95% confidence intervals were provided. In the multivariate model, data were adjusted for demographic characteristics and co-morbid diseases. Abbreviations: APOE, apolipoprotein E; BMI, body mass index; NfL, neurofilament light chain; P-tau181, tau phosphorylated at threonine 181

To further understand the impact of various characteristics and comorbid conditions, an exploratory analysis was conducted. All the participants were categorized into different subgroups according to median age (≥ 70 years/ < 70 years), sex (male/female), APOE genotype (APOE ε4 carrier/non-carrier), median BMI (≥ 24.7 kg/m^2^/ < 24.7 kg/m^2^), median education duration (≥ 12 years/ < 12 years) and medical history of stroke (yes/no), hypertension (yes/no), and diabetes (yes/no). As shown in Fig. 3, consistent correlations between levels of eGFR and plasma NfL were found across all the subgroups. Of note, no significant correlation between levels of eGFR and P-tau181 was found in any subgroup resulting from multivariate models. At the same time, interaction analysis was conducted between various factors and eGFR. To summarize, age demonstrated a significant interaction with eGFR for the impact on plasma NfL (*P*_interaction_ < 0.001), but not for P-tau181. No interactions were found for other factors (*P*_interaction_ > 0.05) (Fig. 3).

**Fig. 4:**
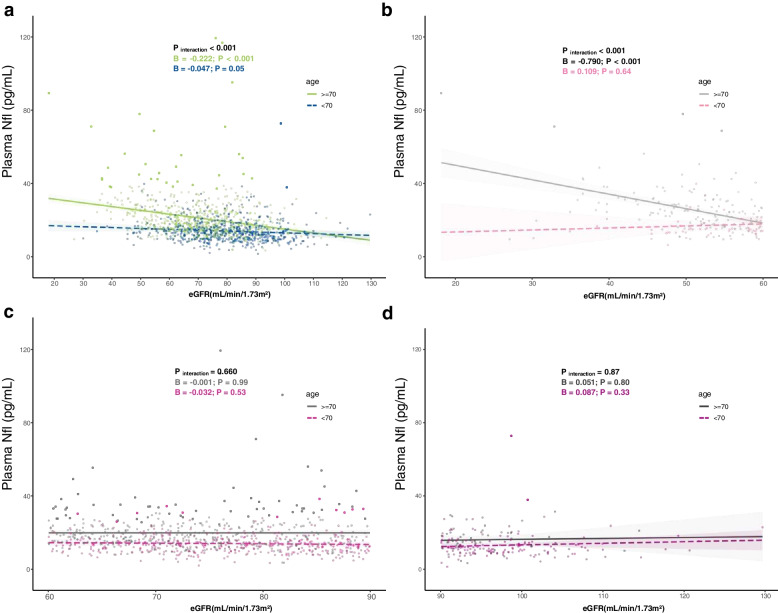
The interaction analysis between age and eGFR. **a** The interaction analysis among age and eGFR. Scatter plots demonstrated whether age modulated the association between plasma biomarkers and eGFR. The green and blue areas represented the 95% confidence interval. **b** The association between eGFR and plasma NfL in different eGFR groups. The pink and grey areas represented the 95% confidence interval. All the models above were adjusted for age, sex, education, BMI, APOE genotype, hypertension, diabetes, and stroke. Abbreviations: NfL, neurofilament light chain; eGFR, estimated glomerular filtration rate

As shown in Fig. 4a, among participants aged ≥ 70 years, a significant negative correlation between eGFR and plasma NfL was observed (*B* = − 0.222, 95% CI − 0.269 to − 0.175, *P* < 0.001). For those aged < 70 years, only a marginally significant correlation was found (*B* = − 0.047, 95% CI − 0.095 to 0.000, *P* = 0.05) (Fig. 4a).

We performed further analysis stratified by three eGFR groups. As shown in Fig. 4b, in those with low eGFR (< 60 mL/min/1.73 m^2^) and aged ≥ 70 years, the correlation between eGFR and plasma NfL was significant (*B* = − 0.790, 95% CI − 1.026 to − 0.554, *P* < 0.001), whereas no significant correlations between other subgroups were observed after adjustment (Fig. 4c and d).

## Discussion

In this cross-sectional study, we assessed the association between renal function and plasma NfL and P-tau181 levels, which yielded three main findings. First, eGFR was negatively associated with plasma NfL and P-tau181. After adjusting for demographic characteristics and comorbid diseases, eGFR remained significantly associated with plasma NfL, but not with P-tau181. Second, a significant interaction between age and eGFR was found on the impact of plasma NfL. Third, in participants ≥ 70 years and with eGFR < 60 mL/min/1.73 m^2^, the correlation between eGFR and plasma NfL was particularly noteworthy.

Blood-based biomarkers are promising in the diagnosis and prognosis of AD [33]. However, biological and technical factors that might negatively affect diagnostic accuracy must be characterized before widespread application [34, 35]. Recent studies showed that renal function might potentially impact blood biomarkers. Elevation of plasma Aβ40, Aβ42, total-tau, NfL, and P-tau181 was demonstrated in patients with CKD [19, 36, 37]. Liu et al. reported that serum Aβ levels were higher in CKD patients, and dialysis CKD patients had lower Aβ levels than non-dialysis ones [36]. Syrjanen et al. found that CKD was associated with higher plasma Aβ40, Aβ42, total-tau, and NfL levels [11]. Sun et al. reported plasma levels of Aβ40, Aβ42, and total-tau were positively associated with the medical history of CKD and negatively associated with eGFR [14]. An investigation by Adam et al. revealed that less than 10% of CKD patients were aware of their disease [38] because CKD remained asymptomatic until the late stage [39]. In previous studies, individuals with renal dysfunction might be underestimated because most diagnoses were based on self-report or medical history. Therefore, we calculated eGFR to reduce reporting bias and enable reliable analysis in a scalable manner.

NfL is released into the cerebrospinal fluid (CSF) and blood upon axon injury [8]. Although being a sensitive indicator for axon damage and neuron death [40], plasma NfL was reported to increase under various conditions other than neurodegenerative diseases. Blood NfL concentration were reported to be correlated with various factors including BMI, age, diabetes, hypertension, dyslipidemia, and renal function [10, 12, 17, 18, 20, 41]. Consistent with these findings, our study found that eGFR was negatively correlated with plasma NfL concentration. There might be some underlying reasons. Since the kidney is responsible for the clearance of most proteins [42], a low eGFR may reduce the clearance of proteins in the blood, and increase the circulating levels for biomarkers, including NfL. This was proposed as the main explanation in prior studies [23, 43]. Kohlhase et al. assessed NfL levels in the serum and urine samples of patients with acute cerebral damage and found that the NfL concentrations in serum and urine were significantly correlated, which indicated that the kidney might play a role in the elimination of peripheral NfL [44]. Meanwhile, elevated NfL levels were found to be associated with cardiovascular diseases, which were often comorbid with CKD [45]. But the association in our study remained significant after adjustment for cardiovascular diseases, i.e., hypertension and diabetes, which suggested other possibilities. Notably, neurological complications were prevalent in patients with renal dysfunction [46]. Accumulation of toxic compounds, including various uraemic compounds, homocysteine, etc., could induce glial dysfunction and neuronal apoptosis, and eventually cause NfL elevation in the blood [47, 48].

Plasma phosphorylated tau including P-tau181, P-tau217, and P-tau231 had been validated as a promising marker for AD [7, 34, 49]. Plasma P-tau181 showed great potential in AD diagnosis [2], cognitive outcome prediction [21], and amyloid and tau PET staging [50]. However, only a few studies had explored the impact of comorbidities on P-tau181. Mielke et al. reported that plasma P-tau level was linked to CKD and other comorbidities [19]. Berry et al. observed patients with cirrhosis and found serum creatinine was negatively associated with P-tau181 [20]. Stocker et al. found that kidney function was negatively associated with P-tau181 [18]. Pan et al. reported that plasma P-tau181 level was positively associated with the medical history of CKD in cognitively normal participants but not in cognitively impaired participants [51]. Janelidze et al. found that CKD and low eGFR were associated with increased plasma phosphorylated tau, but were less associated with phosphorylated tau to total tau ratios [22]. In the current study, although P-tau181 was elevated in the low eGFR group, the difference was not statistically significant after adjusting for confounding factors. The inconsistent result might be related to age, gender, renal function composition, and discrepant calculation for eGFR. Prior studies had demonstrated that plasma P-tau181 specifically reflected AD pathology, and was less related to other pathological conditions, including comorbid diseases [33]. Another possibility was that P-tau181 might be cleared through means other than the kidney, which, however, remained to be elucidated. Albeit preliminary, these findings suggested that P-tau181 was a relatively robust measure that was resilient to biological confounding factors.

Age was one of the most profound risk factors and the main driving force for neurodegeneration. In our study, among different demographic and comorbid characteristics, age was the only modifier on the impact of eGFR on plasma NfL. The eGFR decreased along with aging. Hypertension and diabetes which mainly caused kidney dysfunction were also increasingly prevalent in older adults. Moreover, under chronic stress during aging, cellular senescence was activated with the secretion of various pro-inflammatory molecules, leading to the increment in peripheral markers. Collectively, all the aforementioned mechanisms might contribute to the alteration of plasma NfL and confuse the picture for precise diagnosis. In this study, the correlation of renal function with NfL concentration was significantly remarkable, especially in individuals ≥ 70 years and with eGFR < 60 mL/min/1.73 m^2^. Special attention should be paid to this group when setting up reference ranges of blood-based NfL.

Several advantages of our research should be emphasized. Comprehensive laboratory measures of serum creatinine and cystatin C were assayed and used for the CKD-EPI equation to calculate eGFR, which was superior to other estimations [52, 53]. We used eGFR, rather than the medical history of CKD or other self-reported kidney dysfunction to objectively analyze the relationship between renal function and plasma biomarkers. Additionally, the current study included a large sample of dementia-free individuals from a population-based cohort, which well represented a general older population. Furthermore, establishing the reference ranges of blood biomarkers for the population with diverse ethnicities is warranted for clinical practice. Our findings complemented prior work with valuable data from the Chinese population.

Our findings shall be cautiously interpreted with the following limitations. Firstly, given the cross-sectional study design, we cannot infer a causal relationship between decreased renal function and elevated plasma NfL levels. Secondly, individuals with advanced renal failure were not able to participate in the current study. Most of the participants (83%) had normal or fair renal function (eGFR > 60 mL/min/1.73m^2^), which might underestimate the impact of renal function on plasma biomarkers. However, the eGFR distribution of this study was in accordance with the worldwide CKD prevalence in the general population [54]. Thirdly, only essential demographic and comorbid factors were included in our analysis. There were probably other potential confounders related to renal function and blood biomarkers that might impact their associations. Further, eGFR was calculated by the CDK-EPI equation, however, sensitivity analysis was not conducted utilizing other eGFR equations, including the Lund-Malmö GFR estimating equation [55], which has shown better performance than the CKD-EPI equation in a Swedish population. Moreover, the current study solely examined the association between P-tau 181 and renal function and did not investigate other phosphorylated tau, such as P-tau217 and P-tau 231. Finally, the study participants were from an urban community in a developed metropolis. Whether our findings could represent the population living in other areas remains to be elucidated.

## Conclusion

In summary, the current study demonstrated a negative correlation of eGFR with plasma NfL levels in a population-based cohort. Such correlation was noteworthy in individuals ≥ 70 years and with severe CKD. The results highlighted the importance of considering renal function in interpreting AD biomarkers. Future longitudinal studies should be conducted to analyze the association between renal function and other AD plasma biomarkers with the consideration of more potential confounders and verify the results in different populations. This study contributed to a deeper understanding of the real-world application of AD blood markers.

### Supplementary Information

**Additional file 1: Table S1**. Profile of plasma biomarker levels and neuropsychological test scores in participants with different cognition diagnosis and eGFR levels.** Figure S1**. Correlation between renal function-related indicators and plasma NfL and P-tau181 levels.

## Data Availability

The datasets used and/or analyzed during the current study are available from the corresponding author upon reasonable request.

## Abbreviations

AD: Alzheimer’s disease
NfL: Plasma neurofilament light chain
P-tau181: Phospho-tau181
CKD: Chronic kidney disease
GFR: Glomerular filtration rate
eGFR: Estimated GFR
SAS: Shanghai Aging Study
MCI: Mild cognitive impairment
BMI: Body mass index
APOE: Apolipoprotein E
CKD-EPI: Chronic Kidney Disease Epidemiology Collaboration
IQR: Interquartile range
