# Supplementary material for: The impact of kidney function on plasma neurofilament light and phospho-tau 181 in a community-based cohort: the Shanghai Aging Study
Source: Alzheimers Res Ther. 2024 Feb 12;16:32. doi: 10.1186/s13195-024-01401-2 (PMC10860286; doi:10.1186/s13195-024-01401-2)
Supplement: Supplementary file 1 — Additional file 1: Table S1. Profile of plasma biomarker levels and neuropsychological test scores in participants with different cognition diagnosis and eGFR levels. Figure S1. Correlation between renal function-related indicators and plasma NfL and P-tau181 levels. [file 13195_2024_1401_MOESM1_ESM.docx]

**Additional file 1**

**Table S1. Profile of plasma biomarker levels and neuropsychological test scores in participants with different cognition diagnosis and eGFR levels**

|  | NC_high eGFR | NC_medium eGFR | NC_low eGFR | MCI_high eGFR | MCI_medium eGFR | MCI_low eGFR |
| --- | --- | --- | --- | --- | --- | --- |
|  | n=175 | n=691 | n=161 | n=20 | n=105 | n=37 |
|  | Median (IQR) | Median (IQR) | Median (IQR) | Median (IQR) | Median (IQR) | Median (IQR) |
| Plasma NfL (pg/ml） | 12.04 (9.61, 16.07) | 15.00 (11.58,19.60) | 19.15 (14.89, 25.29) | 14.58 (11.23, 19.10) | 18.33 (14.82, 24.43) | 30 (24.36, 37.83) |
| Plasma p-tau181 (pg/ml) | 1.71 (1.38, 2.18) | 1.87 (1.47, 2.42) | 2.07 (1.59, 2.71) | 2.10 (1.40, 2.63) | 2.05 (1.56, 2.74) | 2.49 (1.83, 3.86) |
| MMSE score | 29.00 (28.00, 30.00) | 29.00 (28.00,30.00) | 29.00 (28.00,30.00) | 28.00 (27.00, 29.00) | 27.50 (25.75, 29.00) | 27.00 (24.75, 28.25) |
| Memory^a^ | 0.27 (-0.22, 0.73) | 0.13 (-0.47,0.73) | 0.03 (-0.41, 0.59) | -1.06 (-1.43, -0.31) | -1.02 (-1.66, -0.59) | -1.66 (-1.9, -0.93) |
| Executive function^a^ | 0.44 (0.08, 0.68) | 0.27 (-0.05, 0.59) | 0.21 (-0.16, 0.56) | -0.26 (-0.53, 0.04) | -0.65 (-1.85, 0.13) | -1.27 (-2.76, -0.16) |
| Attention^a^ | 0.23 (-0.37, 0.89) | 0.67 (-0.53, 0.64) | 0.02 (-0.58, 0.53) | -0.29 (-0.98, 0.39) | -0.74 (-1.06, 0.02) | -0.99 (-1.61, -0.54) |
| Language^a^ | 0.46 (0.08, 0.85) | 0.46 (-0.30, 0.85) | 0.08 (-0.30, 0.85) | 0.08 (-0.88, 0.46) | -0.30 (-1.45, 0.18) | -0.68 (-1.93, 0.18) |
| Visuospatial ability^a^ | 0.06 (-0.75, 0.46) | 0.06 (-0.75, 0.87) | -0.34 (-0.75, 0.87) | -0.75 (-1.15, 0.06) | -0.34 (-1.15, 0.06) | -0.95 (-1.55, -0.34) |

^a^ Z score transformed

Abbreviations: NC, normal control; MCI, mild cognitive impairment; NfL, plasma neurofilament light chain; P-tau181, tau phosphorylated at threonine 181; eGFR, estimated glomerular filtration rate; MMSE, Mini-Mental State Examination.

**Figure S1. Correlation between renal function-related indicators and plasma NfL and P-tau181 levels**

**a**, correlation between renal function-related indicators and plasma NfL levels. The green area represented the 95% confidence interval. **b**, Correlation between renal function-related indicators and plasma P-tau181 levels. The brown area represented the 95% confidence interval.

Abbreviations: NfL, neurofilament light chain; P-tau181, tau phosphorylated at threonine 181; CysC, cystatin C.
